# Inevitable Relative Age Effects in Different Stages of the Selection Process among Male and Female Youth Soccer Players

**DOI:** 10.3390/sports6020029

**Published:** 2018-03-31

**Authors:** Pål Lagestad, Ingebrigt Steen, Terje Dalen

**Affiliations:** Department of Physical Education and Sport Science, Nord University, 7600 Levanger, Norway; ingebrigt91@hotmail.com (I.S.); terje.dalen@nord.no (T.D.)

**Keywords:** soccer, football, adolescents, performance

## Abstract

The relative age effect (RAE) in the selection of young soccer players is a well-known phenomenon. The purpose of this study was to examine the relative age effect existing despite strategies that have been implemented to avoid its presence in the selection process. We also aimed to investigate the RAE during the three different selection stages for B13, B14 (boys), and G13, G14 (girls), and gender differences in the RAE. This was achieved by collecting data from everyone who played soccer in Troendelag, and data that would illuminate the RAE during the three stages of selection for the regional teams of the 2015/2016 season. Mann–Whitney U-tests and Chi-square tests were used as statistical methods. The main finding of this study is that, despite the intention to reduce RAE in the selection process according to the criterion that at least 40% of the players should be born in the second half of the year, both the early-born boys and girls are more likely to be selected. The results also show that the RAE occurs gradually, and the longer the players are in the selection process the more prominent it is. This study highlights the importance of being aware of the RAE when selecting young players.

## 1. Introduction

In Norway, as in many other countries, children and adolescents in sports are grouped based on year of birth. By definition, a range of one year will exist between people who compete in the same age group in sports, such as soccer. The difference in age within the same age group is termed the relative age difference (RAE) [1,2]. Within the same age group, this factor will contribute to individual differences in the rate of development and maturation of physical, technical and mental abilities, in addition with genetic factors, experience, gender, etc. Therefore, the RAE can contribute to advantages and disadvantages for individual achievement in sports [3,4]. The RAE is at its maximum when a child born in late December is compared with another child born in early January of the same year (with January the first as cut off). However, as the children grow, studies have reported that the RAE, as such, will disappear along with growth spurts and the maturation process [1,2]. 

In most sports, those reaching puberty early have an advantage over their later-developing peers, because biological-maturity status constitutes a major factor for performance capacity [5]. In sports in which height, weight, strength and power constitute an advantage, the early-maturing boy or girl at any given age is likely to possess a biological advantage over those who mature later [5]. Therefore, physical maturity in sports gives girls and boys an advantage that might well be mistaken for superior ability. When strict selection procedures and/or criteria are applied to such groups, which is commonly found within sports, RAE should be expected [2]. The individual’s maturation, together with the selection procedures in sports, are suggested to influence the individual’s ability to invest time into practice and to accumulate sport-specific skills and experience, factors that are critical for long-term performance [6].

Theoretical support for the RAE can be found in the concept of developmental-advantage socialisation and the Pygmalion effect [7,8]. Children born early in the year are more often defined as ‘talents’, and are selected for talent camps or teams. The selected children are provided with the best coaches and facilities, and are thus given superior opportunities for development. The initial selection appears, as such, justified because these children develop more quickly than those not selected [9]. Therefore, RAE in sports seems to be enhanced by the so-called Pygmalion effect, in which an individual will perform better when more is expected of him or her (see Rosenthal, 1968). Moreover, Harter’s (1978) competence motivation theory suggests that athletes who perceive that they are able to perform at a high level and are talented, are more likely to continue perfecting their abilities and to invest more time and effort into their sport, with predictable results [8]. Consequently, it may be reasonable to assert that selection for regional teams may affect how adolescents evaluate their talent in soccer. 

The selection procedure for a regional team at B13, G13, B14, and G14 in the middle of Norway comprises three stages. In stage one, the club coaches select the top 2–6 players in the club (approximately 15% of all players) to take part in three training sessions and two matches. Approximately 30–40% of these players are selected, mainly by the club coaches, to participate in stage two, which includes playing two matches. Finally, a regional team coach selects 15% of these players to play for the regional team at stage three. At every stage, everyone involved in the selection process has to select a maximum of 60% of the players born in the first six months of the year, while at least 40% have to be born in the last six months of the year. This is in accordance with the selection document formulated by the Troendelag Regional Football Association (Troendelag fotballkrets, 2016) —a strategy for decreasing RAE in the selection process.

The selection procedures formulated by the Troendelag Regional Football Association are an important strategy in an attempt to prevent RAE in soccer, which has been identified in an extensive body of literature [10,11,12,13,14,15,16,17,18]. However, despite the strategies that have been implemented to avoid RAE in the selection process, it remains unknown if the coaches succeed in avoiding RAE. Studies demonstrate that the RAE decreases with increasing age [12,15,16,19]. However, as in other RAE research [4], most of these studies do not include girls. In fact, only Delorme et al. (2010) and Liu and Liu (2008) include girls, and they documented RAE among French and Chinese girl players, respectively [10,14]. Furthermore, these investigations do not include adolescents younger than 15 years of age, and they do not examine RAE throughout the different stages of the selection process. In general, they focus on RAE in settings of national teams or elite players. 

Knowledge of RAE among young players of both genders, and how it occurs in different stages of the selection process, is critical for coaches that are responsible for selecting players, as well as for researchers, teachers, players, parents, and employers in regional and national soccer associations. However, there is a lack of knowledge in this research area. Therefore, the objective of this study was to elucidate what is the RAE in three selection stages of the regional team (B13, G13, B14, and G14), and when a selection criterion exists of 60/40% born in the first/last six months of the year. We also aimed to identify any gender differences in RAE.

## 2. Materials and Methods

### 2.1. Sample

The data sample is based on register data of 3022 children. These data were anonymized by the Troendelag Regional Football Association. The first group consisted of the number of children born in Norway by month and year (2002 and 2003). In total, this amounted to 111,892 births (see Table 1 below). Data regarding children born in Norway were available (not just Troendelag county), and it was decided to examine the number of live-born in the entire country, as no obvious reason for significant differences should exist between the various regions of Norway. A randomly selected sample of 30 clubs in the Troendelag Regional Football Association comprised of 1812 players (32% of the players, boys and girls, 13 and 14 years of age) were included to determine RAE among all soccer players in this specific age group. The third group represented the initial selection for the regional teams, and consisted of players chosen to participate in training under the auspices of the regional football association (N = 1210). These players were called into three scheduled training sessions and played two matches in player groups of approximately 13 players per team. The fourth group represented the second selection, in which approximately 40% of the players were chosen to advance further (N = 520). The players were assembled into teams (11 field players and two goalkeepers) and played two matches monitored by club coaches, the regional association team coach, and other regional football association staff. The final group consisted of the regional football teams (N = 72). Prior to this selection, a new training session was held, and two matches were played. While club coaches were responsible for the selection to the first two training sessions, the regional football association team coach was responsible for the final selection to the regional football team. The entire selection process took between two and three months. 

### 2.2. Data Collection

In response to our inquiry to Statistics Norway (SSB), we received lists of the number of live-born (2002 and 2003) classified by month and gender in Norway. Anonymised data relating to player gender and date of birth among the 1812 selected players who played soccer for the selected 30 clubs, as well as players selected in the three selection processes, were submitted by the Troendelag Regional Football Association. The project submitted a request for approval to the Data Protection Official for Research (NSD—Norwegian Centre for Research Data, an ethics committee), and permission was granted.

### 2.3. Data Processing and Statistical Analyses

Based on the need for more concrete information relating to the effect of the number of RAE days, the dates of birth of the players were coded from 1 to 365 (1 = 1 January, 365 = 31 December). The results were presented as a median (including percentiles) as a central measure, and interquartile range (IQR) as a measure of dispersion. The data for the dependent variable (date of birth) were skewed right, and not normally distributed. For this reason, the requirements for parametric tests were not satisfied, and non-parametric tests were used. The Mann–Whitney U-test was utilized to highlight differences between boys and girls, as well as year of birth. Chi-square tests was used to examine the association between players born in the first six months versus those born in the last six months, at the second selection and at the regional teams. The significance level was set at p < 0.05. Statistical analyses were performed using SPSS (Statistical Package for the Social Sciences) Statistics 24 (IBM, Armonk, NY, USA).

## 3. Results

**Figure 1:**
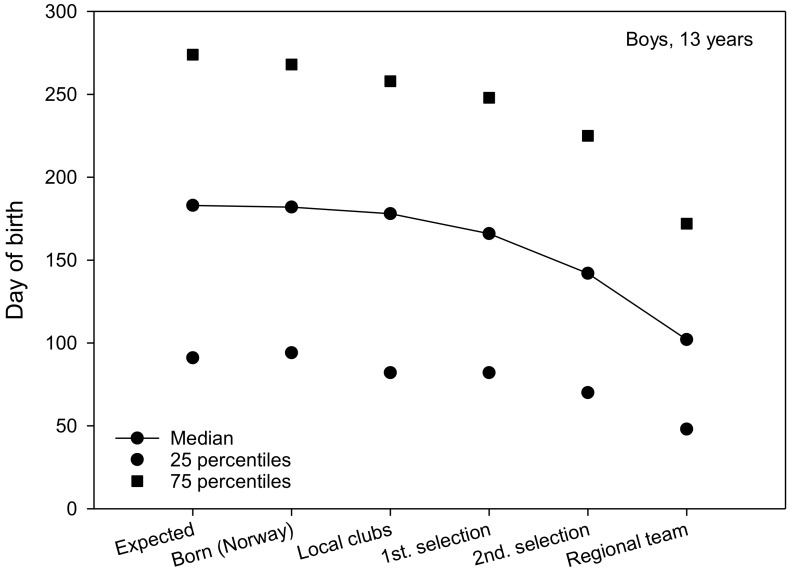
The median date of birth (1 = 1 January, 365 = 31 December) for boys born in 2003 (including the 25th and 75th percentiles).

**Figure 2:**
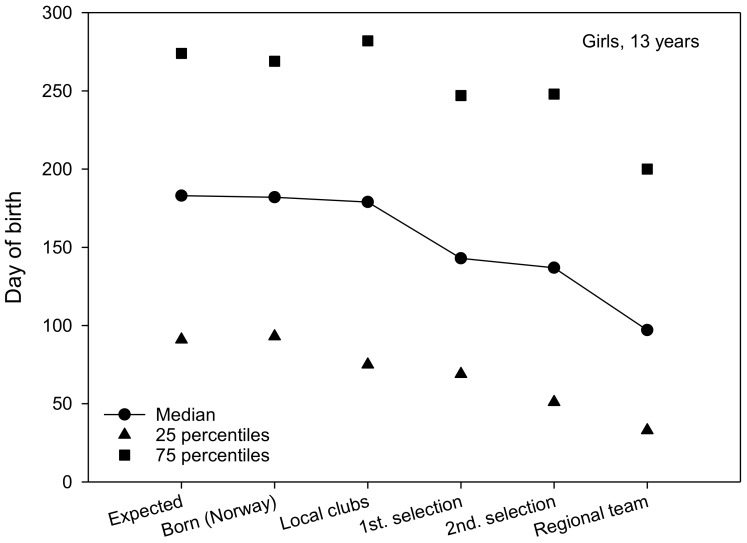
The median for date of birth (1 = 1 January, 365 = 31 December) for girls born in 2003 (including the 25th and 75th percentiles).

The expected date of birth was a constant set at 183 day of birth for all teams. The median for 13-year-old boys and girls was 182 (Figure 1 and Figure 2). The median for local club teams in the Troendelag Regional Football Association was 178 and 179 for 13-year-old boys and girls, respectively. Further in the selection process, the median of the first regional selection was 166 and 143, the second regional selection 142 and 137 and the regional teams 102 and 97, for 13-year-old boys and girls, respectively. For 13-year-old boys, 25% of the regional team were born before 18 February, while 75% of the regional team were born before 22 June (Figure 1). For 13-year-old girls, 25% of the regional team were born prior to 3 February, while 75% of the team were born prior to 20 July (Figure 2). The average regional team player was born approximately two and a half months (76 days) for 13-year-old boys (76 days), and almost three months (82 days) for 13-year-old girls before what might be expected according to the birth data for soccer players in the Troendelag Regional Football Association (Figure 1 and Figure 2). Further analyses revealed that 77.8% of the boys and 72.2% of the girls on the regional team were born in the first six months of the year. Significantly more players were born in the first six months, versus those born in the last six months at the second selection (χ^2^_1_ = 7.2, p = 0.007), and at the regional teams (χ^2^_1_ = 5.3, p = 0.021). 

**Figure 3:**
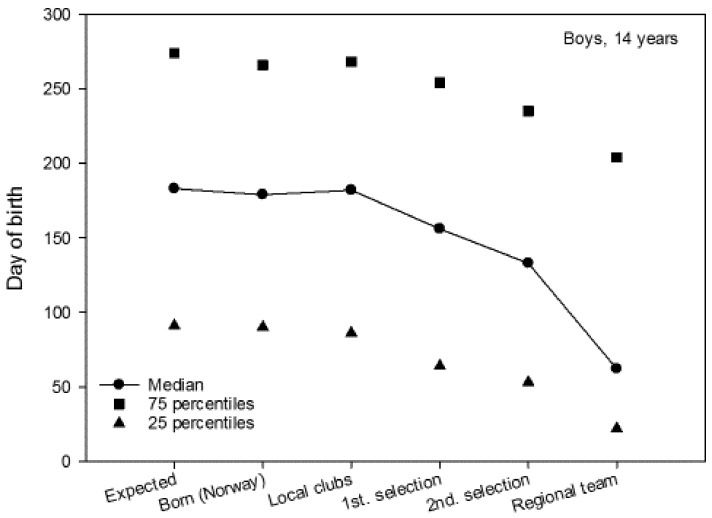
The median for date of birth (1 = 1 January, 365 = 31 December) for boys born in 2002 (including the 25th and 75th percentiles).

**Figure 4:**
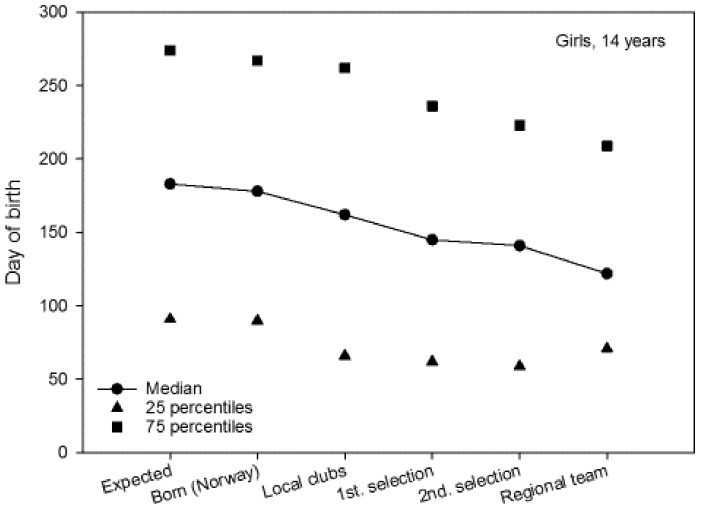
The median for date of birth (1 = 1 January, 365 = 31 December) for girls born in 2002 (including the 25th and 75th percentiles).

The median for 14-year-old boys and girls was 179 and 178, respectively (Figure 3 and Figure 4). The median for local club teams in the Troendelag Regional Football Association was 182 and 162, for 14-year-old boys and girls, respectively. Further in the selection process, the median of the first regional selection was 156 and 145, the second regional selection 133 and 141, and the regional teams 62 and 122, for 14-year-old boys and girls, respectively. For 14-year-old boys, 25% of the regional team were born before 23 January, while 75% of the regional team were born before 24 July (Figure 3). For 14-year-old girls 25% of the regional team were born prior to 13 March, while 75% of the team were born prior to 29 July (Figure 4). The average regional team player was born approximately four months (120 days) for 14-year-old boys, and one and a half months (40 days) for 14-year-old girls before what might be expected according to the birth data for soccer players in the Troendelag Regional Football Association (Figure 3 and Figure 4). Further analyses showed that 72.2% of the 14-year-old boys and 66.7% of the 14-year-old girls on the regional team were born in the first six months of the year. Significantly more players were born in the first six months, versus those born in the last six months at the second selection (χ^2^_1_ = 4.5, p = 0.007), but not at the regional teams (χ^2^_1_ = 1.6, p = 0.215).

Further statistical analyses (Mann–Whitney U-test) revealed that there were no significant gender differences in terms of RAE for club teams (Z = −0.965, p = 0.335), first regional selection (Z = −1.314, p = 0.189), second regional selection (Z = −0.011, p = 0.992), or regional team (Z = −1.014, p = 0.311). Moreover, no significant differences were found in the date of birth between players in the year cohorts 2002 and 2003 in terms of club teams (Z = −0.671, p = 0.502), first regional selection (Z = −1.224, p = 0.221), second regional selection (Z = −0.898, p = 0.369), or regional team (Z= −0.417, p = 0.677). The IQR in three of the four figures shows that the variation in date of birth diminishes the further the players advance in the selection process. Here, G14 stands out by having approximately the same dispersion measure for all six groups. The 25th percentile is also substantially lower for the G14 players.

## 4. Discussion

The main finding of the present study was that, despite the intention to reduce RAE in the selection process, i.e., through setting the criterion that at least 40% of the players should be born in the second half of the year, boys and girls born in the first half of the year are more likely to be selected. In addition, club coaches seem to consider the criterion of distribution (60/40 first/second half of the year) for players in the first selection stage. However, as the competition becomes more intense during the selection process, the distribution criterion becomes increasingly disregarded, and the RAE is highest at the last selection stage conducted by the regional team coaches. At this final stage of the selection process, the RAE among the players is in line with other studies that have investigated RAE in cases in which the distribution criterion is not used for the selection process, i.e., 70–75% born in the first half of the year [2,20]. This is also supported by our finding that the teams for both boys and girls had a higher mean age at each stage throughout the selection process. Musch and Grondin (2001) highlight that competition to obtain a place on the team constitutes one of the primary mechanisms for RAE. That there are representatives from the organisation that set the criteria for distributing players born at different times of the year, and who ultimately select the oldest players, reveals how pervasive RAE is regarding competition between boys and girls in one developmental age group. Indeed, RAE is commonly associated with several major advantages in sports, such as physical capabilities, experience, and psychological factors. 

Children born early in the year often possess the advantage of being taller, stronger, and faster [21]. The results from this study identifying the relationship between date of birth and selection to the regional teams are in accordance with studies in sports, indicating that when advanced physical development is advantageous, the youngest age group is at a relative disadvantage [5]. Moreover, in sports, advanced physical development might be mistaken for superior ability in favour of those who mature early, and therefore are more readily positively evaluated in the selection process. Jimenez and Pain (2008) also argue that such criteria for the selection of young players lead to less development among players who are not selected [22]. 

Another issue that is highlighted by our results is the element of experience. It is suggested that the quantity and quality of practice constitute primary mechanisms in explaining skill or performance attainment [6]. An 11-month difference in age represents almost one year of additional opportunities to practice, which can translate into substantially more training for the oldest pupils. Perhaps a more common factor is that more mature children have been found to have a higher likelihood of being chosen to be part of a team with higher abilities [4]. This will typically provide the more mature adolescents with better coaching, more opportunities to face highly skilled opponents, additional training, and training at a generally higher competition level. Furthermore, more mature children will typically receive the opportunity to have more active participation in the games. 

In Norway, as in many other countries, children and adolescents in both school and sports are grouped based on year of birth. The difference in age within the same age group is called the relative age difference (RAE) [1,2]. Within the same age group, the range of one year between people who compete in the same age group will contribute to individual differences in development and maturation rate. Therefore, the RAE can contribute to advantages and disadvantages for individual achievement in sports and at school [3,4]. Both RAE and biological age should be taken in account for selection. Even if the findings show an increased RAE in the selection process, it is appropriate to note that the results find no RAE among the soccer players who play soccer in Troendelag county, which can be considered to constitute an overall positive situation. 

Another main finding is that no significant gender differences in terms of RAE exist in any of the selection stages. Although several studies have identified RAE in soccer [10,12,13,14,16,17,18,19], most of these studies do not include girls. In fact, only two studies included girls [10,14], and the present findings of RAE among girls support these studies. However, the playing rules in soccer are the same for both boys and girls in terms of physical contact, including tackling, and according to the previous discussion, this equivalence seems to play an important part in creating an RAE. This may explain why the RAE is higher among men than women in ice-hockey [23], where the women’s regulations do not allow as much physical involvement compared to the men’s regulations. 

As argued in the introduction, theoretical support for the RAE is given by developmental-advantage socialisation and the Pygmalion effect [10,11]. Adolescents born early in the year will more often be defined as talents, and be selected to regional teams. Here, they are provided with the best coaches and superior training facilities. With such a strategy, they receive better opportunities for development, and the expectation is fulfilled and justified in relation to the Pygmalion effect [10]. Moreover, athletes who receive such attention from coaches, perceive that they are talented and have a better chance to continue developing their abilities and investing more time in soccer [11].

The present study possesses two major limitations. First, although the overall sample includes 3022 children, the RAE in the four regional teams is analysed with data of only 72 children. In order to increase the robustness of the findings, future work could include a larger sub-sample. Second, only two age groups were examined in the study. Even though these two age groups are considered to be times during which growth spurts are at the highest, future research could incorporate additional age groups to increase the study’s generalizability.

## 5. Conclusions

The main finding of this study is that, despite the intentions to reduce RAE in the selection process, i.e., through introducing the criterion that at least 40% of the players should be born in the second half of the year, boys and girls born in the early part of the year are more likely to be selected. As the competition becomes more intense during the selection process, the distribution criterion is increasingly disregarded, and the RAE is highest at the last selection stage conducted by the regional team coaches. Another main finding is that no significant gender differences in terms of RAE exist in any of the selection stages. We argue that theoretical acknowledgement of the RAE can be found in the concept of developmental-advantage socialisation, and the Pygmalion effect. Knowledge of RAE among young players of both genders, and how it occurs at different stages of the selection process, is critical for coaches who are responsible for selecting players. The study also highlights the importance of being acutely aware of RAE when selecting young players. Further studies should examine interventions, in which the main aim is to determine how to organise regional soccer teams in a way that reduces the practical consequences of RAE in the selection process. 

## Figures and Tables

**Table 1:** Number of participants (N) in the different samples (2015/2016 season).

Group of Players	Boys Born in 2002	Girls Born in 2002	Boys Born in 2003	Girls Born in 2003
Children live-born in Norway in the year cohort	28,325	27,109	29,014	27,444
Total number of players in club teams in Troendelag *	2000	1200	1600	900
Selected players from club teams in Troendelag	551	359	523	379
First selection	337	277	328	268
Second selection	132	130	130	128
Regional team	18	18	18	18

* Number based on estimates from the Troendelag Regional Football Association.

## References

[1] Wattie N., Cobley S., Baker J. (2008). Towards a unified understanding of relative age effects. J. Sports Sci..

[2] Musch J., Grondin S. (2001). Unequal competition as an impediment to personal development: A review of the relative age effect in sport. Dev. Rev..

[3] Baker J.O.E., Schorer J., Cobley S., Schimmer G., Wattie N. (2009). Circumstantial development and athletic excellence: The role of date of birth and birthplace. Eur. J. Sport Sci..

[4] Cobley S., Baker J., Wattie N., McKenna J. (2009). Annual age-grouping and athlete development. Sports Med..

[5] Baxter-Jones A. (1995). Growth and development of young athletes. Should competition levels be age related?. Sports Med..

[6] Baker J., Horton S. (2004). A review of primary and secondary influences on sport expertise. High Abil. Stud..

[7] Rosenthal R., Jacobson L. (1968). Pygmalion in the Classroom: Teacher Expectation and Pupil’s Intellectual Development.

[8] Harter S. (1978). Effectance motivation reconsidered: Toward a developmental model. Hum. Dev..

[9] Rosenthal R., Babad E.Y. (1985). Pygmalion in the gymnasium. Educ. Leadersh..

[10] Delorme N., Boiche J., Raspaud M. (2010). Relative age and dropout in French male soccer. J. Sports Sci..

[11] González-Rodenas J., Calabuig F., Aranda R. (2015). Effect of the game design, the goal type and the number of players on intensity of play in small-sided soccer games in youth elite players. J. Hum. Kinet..

[12] Haulan C., Sather S.A. (2011). Aldersbestemte Fotballandslag i Norge: Dette Kjennetegner de Selekterte Spillerne i 2009.

[13] Helsen W.F., Starkes J.L., Van Winckel J. (1998). The influence of relative age on success and dropout in male soccer players. Am. J. Hum. Biol..

[14] Liu W.-M., Liu D. (2008). A research on the relative age effect among excellent youth female football players in China. J. Beijing Sport Univ..

[15] Mujika I., Vaeyens R., Matthys S.P.J., Santisteban J., Goiriena J., Philippaerts R. (2009). The relative age effect in a professional football club setting. J. Sports Sci..

[16] Sæther S.A. (2016). Presence of the relative age effect and its effect on playing time among under-20 players in the norwegian premier league tippeligaen—A four-year follow up. Monten. J. Sports Sci. Med..

[17] Wiium N., Lie S.A., Ommundsen Y., Enksen H.R. (2010). Does relative age effect exist among Norwegian professional soccer players?. Int. J. Appl. Sports Sci..

[18] Williams J.H. (2010). Relative age effect in youth soccer: Analysis of the FIFA U17 World Cup competition. Scand. J. Med. Sci. Sports.

[19] González-Víllora S., Pastor-Vicedo J.C., Cordente D. (2015). Relative age effect in UEFA championship soccer players. J. Hum. Kinet..

[20] le Gall F., Carling C., Williams M., Reilly T. (2010). Anthropometric and fitness characteristics of international, professional and amateur male graduate soccer players from an elite youth academy. J. Sci. Med. Sport.

[21] Meylan C., Cronin J., Oliver J., Hughes M. (2010). Reviews: Talent identification in soccer: The role of maturity status on physical, physiological and technical characteristics. Int. J. Sports Sci. Coach..

[22] Jimenez I.P., Pain M.T.G. (2008). Relative age effect in Spanish association football: Its extent and implications for wasted potential. J. Sports Sci..

[23] Wattie N., Baker J., Cobley S., Montelpare W.J. (2007). A historical examination of relative age effects in Canadian hockey players. Int. J. Sport Psychol..

